# Role of cesarean section in the development of neonatal gut microbiota: A systematic review

**DOI:** 10.1515/med-2021-0270

**Published:** 2021-04-09

**Authors:** Negin Shaterian, Fatemeh Abdi, Nooshin Ghavidel, Farzane Alidost

**Affiliations:** Student Research Committee, Nursing and Midwifery faculty, Shahid Beheshti University of Medical Sciences, Tehran, Iran; Non-communicable Diseases Research Center, Alborz University of Medical Sciences, Karaj, Iran; Social Determinants of Health Research Center, Alborz University of Medical Sciences, Karaj, Iran; Student Research Committee, School of Nursing and Midwifery, Tehran University of Medical Sciences, Tehran, Iran

**Keywords:** gut microbiome, neonate, cesarean section, vaginal delivery

## Abstract

**Background:**

The delivery mode is one of the factors affecting the type of colonization of the human gut. Gut colonization affects all stages of the human life cycle, and the type of gut microbiome can contribute to immune system function, the development of some diseases, and brain development; and it has a significant impact on a newborn’s growth and development.

**Methods:**

Terms defined as MeSH keywords were searched by the databases, and web search engines such as PubMed, ClinicalTrials.gov, Embase, Scopus, ProQuest, Web of Science, and Google Scholar were searched between 2010 and 2020. The quality of each study was assessed according to the Newcastle–Ottawa scale, and seven eligible and high-quality studies were analyzed.

**Finding:**

The abundances of *Bacteroides* and *Bifidobacterium* during the first 3 months of life; *Lactobacillus* and *Bacteroides* during the second 3 months of life; *Bacteroides* and *Bifidobacterium* during the second 6 months of life; and *Bacteroides*, *Enterobacter*, and *Streptococcus* after the first year of life were higher in vaginal delivery-born infants. While infants born by cesarean section (CS) had higher abundances of *Clostridium* and *Lactobacillus* during the first 3 months of life, *Enterococcus* and *Clostridium* during the second 3 months of life, and *Lactobacillus* and *Staphylococcus* after the first year of life.

**Discussion:**

Delivery mode can affect the type of the human intestinal microbiota. The CS-born babies had lower colonization rates of *Bifidobacterium* and *Bacteroides*, but they had higher colonization rates of *Clostridium*, *Lactobacillus*, *Enterobacter*, *Enterococcus*, and *Staphylococcus*. Given the effect of microbiota colonization on neonatal health, it is therefore recommended to conduct further studies in order to investigate the effect of the colonization on the delivery mode and on baby’s growth and development.

**Application to practice:**

The aim of this study was to investigate the role of CS in the development of the neonatal gut microbiota.

## 1 Background

Abundant microbes associated with humans can form microbial communities called the human microbiota [1]. The neonatal gut microbiota colonization seems to be important for his or her health and development because the developing infant gut microbiome can influence metabolism, immune system function, and brain development [2]. When the initial colonization occurs in the beginning of infancy and adulthood, the microbiome can be influenced by several factors including genetics, maternal prenatal stress [3], culture [4], delivery mode, use of antibiotics, nutrition, environment, health, and disease status [5]. The newborns’ gastrointestinal tract is sterile, but it becomes colonized immediately after birth with the bacteria from the environment, mainly from the mother [6], and the microbiota of an infant can develop rapidly after birth [7]. However, recent studies have shown that microbial accumulation occurs in the uterus [8] and continues to start accumulating intestinal microbiota until a relatively stable state is reached [9]. The process of early intestinal colonization can vary greatly from person to person and is influenced by several factors such as the mode of delivery that can play an important role in the early establishment of gut microbiota and the newborn’s immune system [10]. Birth by cesarean section (CS) and insufficient breastfeeding have been reported to induce an abnormal gut microbiome composition in infants’ gut and may also lead to increased risk of several serious health conditions in children, including asthma and allergies, celiac disease, diabetes, and obesity [11], which may be due to reduced exposure to maternal microbes during birth [12]. Early colonization patterns can be influenced by delivery mode and even types of CS because the patterns can differ based on the delivery by means of elective or emergency CS [13]. Infants born by vaginal delivery (VD), unlike those born by CS, are mostly colonized with the maternal vaginal and intestinal flora [14], and these differences seem to be present during infancy [15]. Early gut microbiota may affect subsequent microbiota [16]. Studies conducted on the neonatal gut microbiota have been restricted to culture-based enumeration, 16S-based profiling, and/or small sample sizes [17]. Over the past few decades, there has been a steady rise in the rate of CS delivery worldwide in spite of the absence of any medical indications [18]. In some situations if there is no evidence in favor of CS such as mothers infected with COVID-19, the type of delivery should be based on the usual obstetric indications and maternal requests [19]. In some countries, more than 50% of births occurs by CS, and more than 15% of all women give birth by CS for the protection of the health of both themselves and their babies [20]. The gut microbiota is a highly complex ecosystem containing 10^14^ bacteria, and there are approximately 160 such species in the fecal samples of each individual [21]; and its genome, which is guessed to be 100 times greater than that of human genome, can be defined as a microbiome [22] and the number of its bacteria is 10 times more than the total number of human cells, especially after the bacterial colonization of the infant. The gut microbial composition is unique for each individual although more than 95% can be assigned to one of four major phyla: Firmicutes, Bacteroidetes, Actinobacteria, and Proteobacteria [23]. Knowing the patterns of microbial intestinal colonization of healthy infants based on determining the effects of specific health and changeable risk factors are crucial in the early years of life [24].

### 1.1 Evidence-based practice purpose

Given that the colonization of the neonatal gut microbiome is influenced by several factors including the mode of delivery, therefore, the aim of this study was to investigate the role of CS in the development of the neonatal gut microbiota.

## 2 Methods

The guidelines of Preferred Reporting Items for Systematic Review and Meta-Analysis (PRISMA) were followed while reporting the study protocol [25,26]. Also, in accordance with the PRISMA guidelines, the following steps were taken: a systematic literature search, organization of documents for the review, abstracting and quality assessment of each empirical study, synthesizing data, and writing the report [27].

### 2.1 Search strategy

In this systematic review, the databases and web search engines such as Google Scholar, PubMed, ClinicalTrials.gov, Embase, Scopus, ProQuest, and Web of Science were searched between 2010 and 2020. In addition, we searched according to MeSH as well (Table 1).

**Table 1:** Search strategies for systematic review

1.	“Cesarean Sections” [MeSH] OR “Delivery, Abdominal” [MeSH] OR “Abdominal Deliveries” [MeSH] OR “Deliveries, Abdominal” [MeSH] OR “Caesarean Section” [MeSH] OR “Caesarean Sections” [MeSH] OR “Abdominal Delivery” [MeSH] OR “C-section (OB)” [MeSH] OR “C Section (OB)” [MeSH] OR “C-sections (OB)” [MeSH]
2.	“Gastrointestinal Microbiomes” [MeSH] OR “Microbiome, Gastrointestinal” [MeSH] OR “Gut Microbiome” [MeSH] OR “Gut Microbiomes” [MeSH] OR “Microbiome, Gut” [MeSH] OR “Gut Microflora” [MeSH] “Gut Microbiota” [MeSH] OR “Gut Microbiotas” [MeSH] OR “Microbiota, Gut” [MeSH] OR “Gastrointestinal Flora” [MeSH] OR “Flora, Gastrointestinal” [MeSH] OR “Gut Flora” [MeSH] OR “Flora, Gut” [MeSH] OR “Gastrointestinal Microbiota” [MeSH] OR “Gastrointestinal Microbiotas” [MeSH] OR “Microbiota, Gastrointestinal” [MeSH] OR “Gastrointestinal Microbial Community” [MeSH] OR “Gastrointestinal Microbial Communities” [MeSH] OR “Microbial Community, Gastrointestinal” [MeSH] OR “Gastrointestinal Microflora” [MeSH] OR “Microflora, Gastrointestinal” [MeSH] OR “Gastric Microbiome” [MeSH] OR “Gastric Microbiomes” [MeSH] OR “Microbiome, Gastric” [MeSH] OR “Intestinal Microbiome” [MeSH] OR “Intestinal Microbiomes” [MeSH] OR “Microbiome, Intestinal” [MeSH] OR “Intestinal Microbiota” [MeSH] OR “Intestinal Microbiotas” [MeSH] OR “Microbiota, Intestinal” [MeSH] OR “Intestinal Flora” [MeSH] OR “Flora, Intestinal” [MeSH] OR “Enteric Bacteria” [MeSH] OR “Bacteria, Enteric” [MeSH]
3.	“Infant” [MeSH] OR “Infants” [MeSH] OR “Newborn” [MeSH]
4.	#1 AND #2 AND #3

### 2.2 Inclusion criteria

#### 2.2.1 Types of studies

Cohorts and cross-sections conducted between 2010 and 2020 were included in this review. Letters, comments, controlled trials, randomized-controlled clinical trials, and quasi-experimental and observational studies, as well as case reports, were excluded. There are no language restrictions to use and enter articles in this study. If the language used in an article is other than Persian or English, we asked a translator to translate the article.

#### 2.2.2 Types of participants

The studies were selected if:

– their participants were healthy full-term infants;
– bacteria found in the gut microbiota had no restriction;
– the studies investigated *Clostridium*, *Bacteroides*, *Bifidobacterium*, *Lactobacillus*, *Enterobacter*, *Streptococcus*, and *Enterococcus.*

#### 2.2.3 Types of interventions

The studies were reviewed if studies mentioned the percentages/means of each gut microbiota colonization in each stage of life (after birth to after 1 year).

#### 2.2.4 Types of outcome measure

The method to identify and detect the bacteria is summarized in Table 3.

#### 2.2.5 Study selection

The titles and abstracts of articles and the eligible studies were first reviewed. Then two authors independently reviewed the full text of articles and they discussed discrepancies until agreement was reached. Afterward, a table was prepared by reviewing several articles that best reflect the data of each article in order to make a decision by collecting data from articles on CS and neonatal gut microbiota.

### 2.3 Quality assessment

The quality of each study was assessed according to the Newcastle–Ottawa scale (NOS) [28]. A maximum of ten stars can be given to each study based on the NOS. A maximum of five stars can be given to the selection (such as sample size, nonrespondents, and ascertainment of the exposure). A maximum of two stars can be given to the comparability (such as the study control for the most important factor). A maximum of three stars can be given to the outcome (such as assessment of the outcome and statistical test). Studies of high-quality score nine or ten stars, and studies with a score of seven or eight stars are considered to be of medium quality, and also studies scoring less than six stars are considered to be of low quality [29]. The quality score for each article is summarized in Table 2.

**Table 2:** Overview of all included studies in systematic review

Reference	Study type	Location	Delivery mode (number)		Gestational age (week)	Feeding type	Use of antibiotics	Sample collection time	Quality score
			CS	VD					
Lee [32]	Cross-sectional	Korea	3	3	Not reported	A combination of breastfeeding formula feeding	No use of antibiotics	1–3 days	8
								1 month after birth	
								6 months after birth	
Nagpal [33]	Cohort	Japan	17	134	38	Exclusive breastfeeding	Antibiotic is given to 3 VD-born infants	24–48 h after birth	9
								3–7 days after birth	
								1, 3, and 6 months after birth	
								3 years after birth	
Martin et al. [2]	Cross-sectional	Belgium	28	80	<37	Exclusive breastfeeding; a combination of breastfeeding formula feeding	Antibiotics is given to 6 infants	the first bowel movement	8
								2 days after the first bowel movement	
								1 week after birth	
								1, 3, and 6 months after birth	
								1 week after stopping breastfeeding	
Azad [34]	Cohort	Canada	43	155	38	Exclusive breastfeeding, partial breastfeeding	No use of antibiotics in infants,	3 months after birth	7
							No use of antibiotic prophylaxis in 96 women during the delivery,	1 year after birth	
							Use of antibiotic prophylaxis in 102 women during the delivery		
Madan et al. [24]	Cohort	United States	32	70	<37	Exclusive breastfeeding; exclusive formula feeding; a combination of breastfeeding formula feeding		6 weeks after birth	8
Liu [35]	Cross-sectional	China	16	25	38–40	A combination of breastfeeding formula feeding;	No use of antibiotics in infants	2 days after birth	8
						Use of antibiotic prophylaxis in women prior to cesarean section		4 days after birth	
Azad [36]	Cohort	Canada	6	18	37–41	9 babies not breastfed, 5 babies breastfed partially, and 10 babies breastfed exclusively	19 infants not used antibiotics, not to mention the use of antibiotics for 2 babies who were given ampicillin and gentamicin to 1 VD-born infant (2 days), amoxicillin is given to 2 CS-born infants (6–12 weeks), 12 women not use the antibiotics, 1 woman did not use antibiotic seriously, ampicillin is given to 1 woman after delivery, during 20 weeks of gestation azithromycin penicillin G are given to 1 woman,	3–4 months after birth	8
							Cephalexin is given to one woman during the 31st week of pregnancy,		
							Cefazolin is given to 2 women prior to cesarean section,		
							Clindamycin is given to 1 woman prior to cesarean section,		
							Penicillin G is given to three women,		
							Cephalexin is given to 1 woman after cesarean section,		
							Cefazolin and metronidazole are given to 1 woman after cesarean section		

### 2.4 Data extraction

Two investigators independently searched for relevant scientific publications, carried out validity assessments [30], and any disagreements were resolved [31]. The demographic data of each selected article such as reference, study type, location, delivery mode (number), gestational age (week), feeding type, use of antibiotics, sample collection, and quality score are summarized in Table 2. In addition, the method to identify and detect the bacteria, sampling time (days), and microbiota (mean/number) % are summarized in Table 3.

**Table 3:** The diversity and colonization rates of neonatal gut microbiota

Author	The method to identify and detect the bacteria	Sampling time (days)	Microbiota (mean/number) %															
			*Clostridium*		*Bacteroides*		*Bifidobacterium*		*Lactobacillus*		*Staphylococcus*		*Enterobacter*		*Streptococcus*		*Enterococcus*	
			NVD	CS	NVD	CS	NVD	CS	NVD	CS	NVD	CS	NVD	CS	NVD	CS	NVD	CS
Lee [32]	16SrRNA gene analysis performed by 454 pyrosequencing of the V1–V3 regions	From birth to the 7th day	0.2	0.0	11.9	0.0	0.6	0.3	0.3	33.3	1.0	18.7	NR*	NR	NR	NR	NR	NR
		8–30	0.0	33.1	0.4	1.2	24.3	1.1	4.4	4.1	4.09	0.007						
		91–180	0.1	31.2	0.0	1.8	2.6	0.4	0.0	0.0	0.007	0.002						
Nagpal [33]	RNA Extraction and RT-qPCR	After the 1st year of life	4.0 ± 0.9	3.9 ± 0.6	5.3 ± 2.0	5.0 ± 2.3	5.7 ± 1.7	5.7 ± 1.6	3.7 ± 0.9	5.0	5.4 ± 1.5	5.9 ± 1.7	7.0 ± 1.8	5.9 ± 1.6	4.4 ± 1.2	3.9 ± 0.6	5.0 ± 1.8	4.9 ± 1.8
Martin et al. [2]	DNA was extracted from the PBS-suspension mentioned above and subjected to qPCR	From birth to the 7th day	12.8	23.2	23.6	0.2	41.8	11.7	30.9	11.7	NR	NR	NR	NR	NR	NR	52.2	49.3
		8–30	24.1	53.6	33.9	3	61.5	35.7	34.1	33.9							72.2	92.9
		31–90	33.3	35.7	40.0	15.4	69.8	65.1	31.4	35.7							89.7	96.5
		91–180	36.4	34.6	46.2	26.2	77.3	79.8	28.6	23.0							97.4	100
Azad [34]	DNA extraction and amplification Illumina 16S rRNA sequencing, and taxonomic classification	31–90	0.5	1.1	29.4	0.2	5.0	5.8	NR	NR	NR	NR	16.6	27.9	NR	NR	NR	NR
		181–360	0.1	0.2	50.2	42.2	2.0	1.3					1.0	1.2				
Madan et al. [24]	next-generation sequencing of the 16S rRNA gene	31–90	5.1	8.8	34.6	20.7	23.3	17.4	2.5	4.2	1.6	3.4	NR	NR	12.1	14.0	4.3	8.7
Liu [35]	polymerase chain reaction (PCR)–denaturing gradient gel electrophoresis in combination with 16S ribosomal RNA (rRNA) gene sequencing of the clones corresponding to the degenerating gradient gel electrophoresis (DGGE) bands	From birth to the 7th day	0.0	5.7	8.2	2.8	1.9	0.0	1.5	1.5	0.0	4.8	3.9	3.0	0.6	5.1	0.7	2.3
Azad [36]	signature gene used was 16S rRNA	From birth to the 7th day	2.8 ± 2.0	2.1 ± 1.0	1.0 ± 0.4	0.0 ± 0.0	36.6 ± 7.8	48.6 ± 14.8	NR	NR	NR	NR	13.7 ± 2.7	6.2 ± 3.1	4.7 ± 2.4	8.7 ± 6.8	1.6 ± 0.6	0.9 ± 0.8

^*^NR: not reported.

### 2.5 Eligible criteria

The study inclusion criteria were as follows: all studies published in English between 2010 and 2020 in which healthy full-term infants were examined, and bacteria found in the gut microbiota had no restriction. The studies investigating *Clostridium*, *Bacteroides*, *Bifidobacterium*, *Lactobacillus*, *Enterobacter*, *Streptococcus*, and *Enterococcus* and studies that mentioned the percentages/means of each gut microbiota colonization in each stage of life (after birth to after 1 year) were included in the study. The publications, such as reviews, letters, comments, and case reports, studies in which the difference between VD-born infants and CS-born infants were expressed as the number of clones (but not as percentages/means), and studies examining the effects of delivery mode on bacterial colonization leading to a specific disease in the newborn were excluded from the study.

### 2.6 Findings

**Figure 1:**
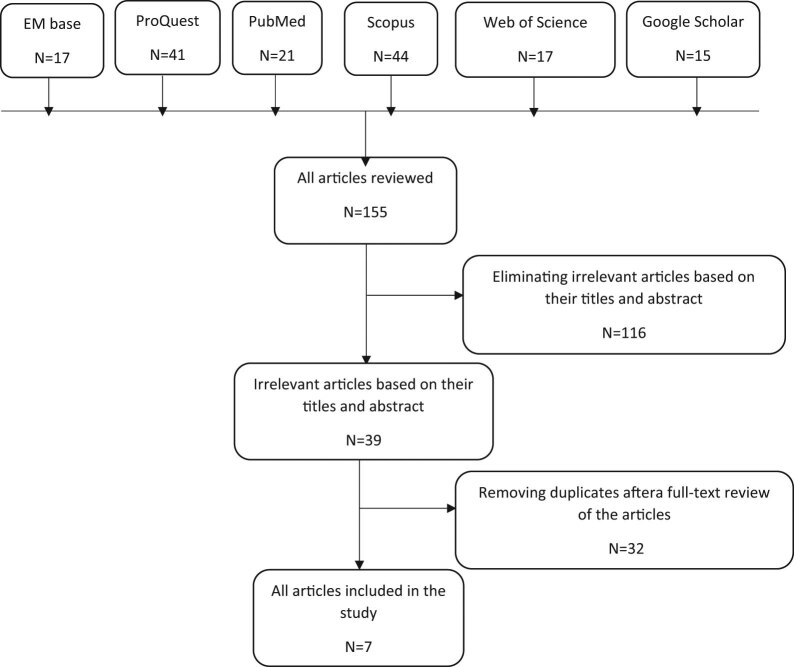
Search flow diagram.

This study was reported based on the PRISMA guidelines [37]. The systematic search in the databases identified 155 articles. After reviewing their titles and abstracts, 116 irrelevant articles and 32 full-text articles due to duplication were removed. Finally, seven articles were included in the systematic review. Flowchart of studies included in the review is shown in Figure 1. The characteristics of included studies are presented in Table 1 and their main findings are summarized in Table 2. In the order of frequency, the countries where the articles were published were Canada, the United States, Korea, Japan, Belgium, and China.

**Figure 2:**
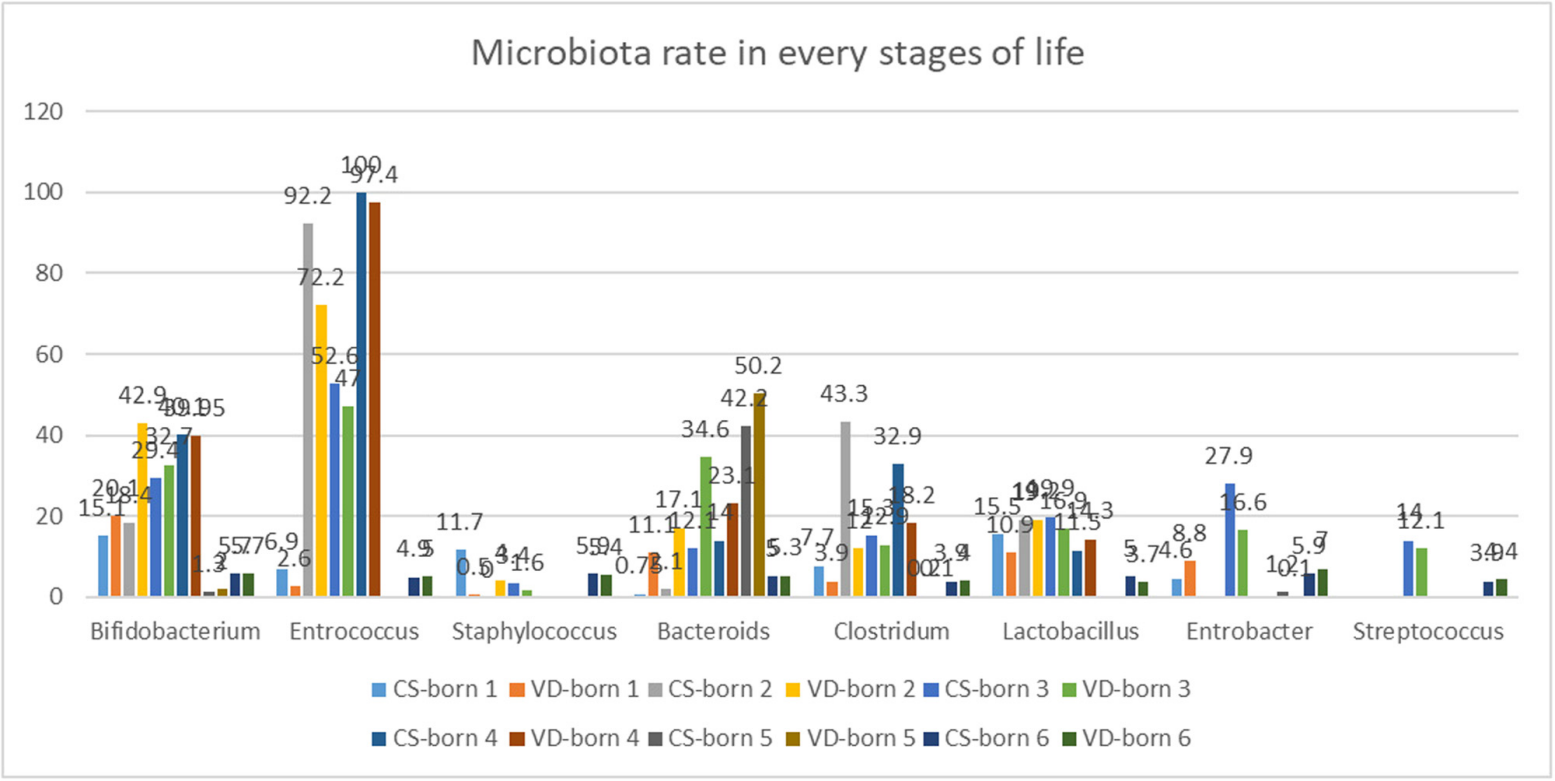
The total colonization rates and gut microbiota colonization rates in each time. CS-born 1 and VD-born 1: 1st week, CS-born 2 and VD-born 2: 8 to 30 days, CS-born 3 and VD-born 3: 31 to 90 days, CS-born 4 and VD-born 4: 91 to 180 days, CS-born 5 and VD-born 5: 181 days to 1st year, CS-born 6 and VD-born 6: after 1st year.

### 2.7 Factors examined in the studies

The factors studied in the studies included the type of study, country, number of participants in the VD and CS groups, gestational age, feeding type, antibiotic use in mother and infant, sample collection time, and the quality score of each article. There was a difference in the included studies with respect to the infant feeding type. Three studies showed antibiotic use in infants, and another three studies indicated antibiotic use in a number of mothers. Sample collection time varied from birth to 3 years after birth in the included studies.

### 2.8 Neonatal gut microbiota

The neonatal gut microbiota examining in the included studies were as follows: *Bifidobacterium*, *Bacteroides*, *Clostridium*, *Lactobacillus*, *Enterobacter*, *Enterococcus*, and *Staphylococcus*. Table 3 shows the rates of neonatal gut microbiota and their mean according to the following schedule: the first week of life, during 8 days to 1 month of life, during 31 days to 3 months of life, during 91 days to 6 months of life, during 181 days to 1 year of life, and after 1 year of life.

### 2.9 The diversity and colonization rates of neonatal gut microbiota during the first week of life

Colonization rate of gut microbiota in CS-born infants was lower than that in VD-born infants in their first week of life and their means were 10.0 and 10.5, respectively. The colonization rate of neonatal gut microbiota during the first week of life was explored in four studies. The highest rate of colonization of VD-born infants was related to *Bifidobacterium* (mean = 20.1), and the highest rate of the colonization of CD-born infants was related to *Enterococcus* (mean = 17.5). Also, the lowest rates of colonization of CD-born infants and VD-born infants were related to *Staphylococcus* and *Bacteroides* and their colonization means were 0.5 and 0.7, respectively. Significant differences were observed between the two groups with respect to the mean colonization rates of *Clostridium* (VD group = 3.9 and CS group = 7.7), *Bacteroides* (VD group = 11.1 and CS group: 0.75), *Bifidobacterium* (VD group = 20.1 and CS group = 15.1), *Lactobacillus* (VD group = 10.9 and CS group = 15.5), *Staphylococcus* (VD group = 0.5 and CS group = 11.7), *Enterobacter* (VD group = 8.8 and CS group = 4.6), and *Enterococcus* (VD group = 2.6 and CS group = 6.9). The colonization rates of *Bifidobacterium*, *Bacteroides*, *Enterobacter*, and *Enterococcus* were higher in the VD group, while the colonization rates of *Clostridium*, *Lactobacillus*, *Staphylococcus*, and *Streptococcus* were higher in the CD group (Figure 2).

### 2.10 The diversity and colonization rates of neonatal gut microbiota during 8 days to 1 month of life

According to the assessed articles and the mean of gut microbiota rate in both studies that mentioned the rate of gut microbiota during 8 days to 1 month, no significant difference was found between CS and VD groups with respect to colonization rates of gut microbiota during their second week of life to 1 month of life and its mean was 25.8. The colonization rate of neonatal gut microbiota during 8 days to 1 month of life was investigated in two studies. The highest rate of colonization of the VD group was related to *Bifidobacterium* (mean = 42.9), and also the highest rate of the colonization of the CS group was related to *Enterococcus* (mean = 92.9). Moreover, the lowest rates of colonization of VD and CS groups were related to *Staphylococcus* and their colonization means were 4.0 and 0.0, respectively. There have been no reports of the colonization rates of *Enterobacter* and *Streptococcus* in the included studies. No statistically significant difference was observed between the two groups in terms of the mean colonization rate of *Lactobacillus* (VD group = 19.2 and CS group = 19). However, there were significant differences between the two groups with respect to the mean colonization rates of *Clostridium* (VD group = 12.0 and CS group = 43.3), *Bacteroides* (VD group = 17.1 and CS group = 2.1), *Bifidobacterium* (VD group = 42.9 and CS group = 18.4), *Staphylococcus* (VD group = 4.0 and CS group = 0.0), and *Enterococcus* (VD group = 72.2 and CS group = 92.9). The colonization rates of *Bifidobacterium*, *Bacteroides*, and *Staphylococcus* were higher in the VD group, while the colonization rates of *Clostridium* and *Enterococcus* were higher in the CS group.

### 2.11 The diversity and colonization rates of neonatal gut microbiota during 31 days to 3 months of life

Colonization rate of gut microbiota in CS-born infants was lower than that in VD-born infants during their second month of life to 3 months of life and their means were 22.5 and 24.9, respectively. The diversity and colonization rates of neonatal gut microbiota during 31 days to 3 months of life were examined in the three studies. Furthermore, the highest rates of colonization of VD and CS groups were related to *Enterococcus* and their colonization means were 52.6 and 47.0, respectively. Also, the lowest rates of colonization of the VD and CS groups were related to *Staphylococcus* and their colonization means were 3.4 and 1.6, respectively. There were significant differences between the two groups with respect to the mean colonization rates of *Clostridium* (VD group = 12.9 and CS group = 15.3), *Bacteroides* (VD group = 34.6 and CS group = 12.1), *Bifidobacterium* (VD group = 32.7 and CS group = 29.4), *Lactobacillus* (VD group = 16.9 and CS group = 19.9), *Staphylococcus* (VD group = 1.6 and CS group = 3.4), *Enterobacter* (VD group = 16.6 and CS group = 27.9), *Streptococcus* (VD group = 12.1 and CS group = 14.0), and *Enterococcus* (VD group = 47 and CS group = 52.6). The colonization rates of *Bifidobacterium*, *Bacteroides*, and *Staphylococcus* were higher in the VD group, while the colonization rates of *Clostridium*, *Lactobacillus*, *Enterobacter*, *Streptococcus*, *Enterococcus*, and *Staphylococcus* were higher in the CS group.

### 2.12 The diversity and colonization rates of neonatal gut microbiota during 91 days to 6 months of life

Colonization rate of gut microbiota in VD-born infants was lower than that in CS-born infants during their third to 6 months of life and their means were 28.8 and 29.7, respectively. The diversity and colonization rates of neonatal gut microbiota during 91 days to 6 months of life were explored in two studies. Furthermore, the highest rates of colonization of the VD and CS groups were related to *Enterococcus* and their colonization means were 100 and 97.4, respectively. Also, the lowest rates of the colonization of the VD and CS groups were related to *Staphylococcus*, and their colonization means were 0.007 and 0.002, respectively. There have been no reports of the colonization rates of *Enterobacter* and *Streptococcus* in the included studies. No significant difference was observed between the two groups in terms of the total mean colonization rates of *Bifidobacterium* (VD group = 39.95 and CS group = 40.1). As mentioned earlier, no significant difference was found between the two groups with respect to the total mean colonization rate of *Staphylococcus*. Significant differences were observed between the two groups with respect to the mean colonization rates of *Clostridium* (VD group = 18.2 and CS group = 32.9), *Bacteroides* (VD group = 23.1 and CS group = 14), *Lactobacillus* (VD group = 14.3 and CS group = 11.5), and *Enterococcus* (VD group = 97.4 and CS group = 100). The colonization rates of *Bacteroides* and *Lactobacillus* were higher in the VD group, while the colonization rates of *Clostridium* and *Enterococcus* were higher in the CS group.

### 2.13 The diversity and colonization rates of neonatal gut microbiota during 181 days to 1 year of life

Colonization rate of gut microbiota in CS-born infants was lower than that of VD-born infants during their 7th month to 1 year of life and their means were 11.2 and 13.3, respectively. The diversity and colonization rates of neonatal gut microbiota from 181 days to 1 year of age were investigated in one study. In addition, the highest rates of colonization of the VD and CS groups were related to *Bacteroides* and their colonization means were 50.2 and 42.2, respectively. Also, the lowest rate of colonization of the VD-born infants was related to *Clostridium* (mean = 0.1), and the lowest rate of the colonization of CS-born infants was related to *Clostridium* (mean = 0.2). There have been no reports of the colonization rates of *Lactobacillus*, *Staphylococcus*, *Streptococcus*, and *Enterococcus* in the included study. No statistically significant differences were observed between two groups with respect to the mean colonization rates of *Clostridium* (VD group = 0.1 and CS group = 0.2) and *Enterobacter* (VD group = 0.1 and CS group = 1.2), but statistically significant differences were observed between two groups in terms of the mean colonization rates of *Bacteroides* (VD group = 50.2 and CS group = 42.2) and *Bifidobacterium* (VD group = 2 and CS group = 1.3). Additionally, the colonization rates of *Bacteroides* and *Bifidobacterium* in the VD group were higher than those in the CS group.

### 2.14 The diversity and colonization rates of neonatal gut microbiota after 1 year of life

Colonization rate of gut microbiota in VD-born infants was marginally higher than that in the CS-born infants after their first year of life and their means were 5.02 and 5.06, respectively. The colonization rate of neonatal gut microbiota after 1 year of life was examined in one study. The highest rate of colonization of VD-born infants was related to the *Enterobacter* (mean = 42.9), and also the highest rates of the colonization of CS-born infants were related to the *Staphylococcus* and *Enterobacter* and their means were 5.9 and 5.9, respectively. The lowest rate of colonization of the VD-born infants was related to *Lactobacillus* (mean = 3.7), and the lowest rates of the colonization of the CS-born infants were related to *Clostridium* and *Streptococcus* (mean = 3.9). No statistically significant differences were observed between two groups with respect to the mean colonization rates of *Bifidobacterium* (in both the groups = 5.7) and *Clostridium* (VD group = 4.0 and CS group = 3.9) but statistically significant differences were observed between two groups in terms of the mean colonization rates of *Bacteroides* (VD group = 5.3 and CS group = 5.0), *Lactobacillus* (VD group = 3.7 and CS group = 5.0), *Staphylococcus* (VD group = 5.4 and CS group = 5.9), *Enterobacter* (VD group = 7.0 and CS group = 5.9), *Enterococcus* (VD group = 5.0 and CS group = 4.9), and *Streptococcus* (VD group = 4.4 and CS group = 3.9). Furthermore, the colonization rates of *Bacteroides*, *Enterobacter*, and *Streptococcus* were higher in those born by VD, while the colonization rates of *Lactobacillus* and *Staphylococcus* were higher in CS-born infants. All these bacteria colonized the gut of VD-born infants in their first year of life and *Clostridium* colonized the gut of the CS-born infants.

## 3 Discussion

The results of the present study showed that the diversity and colonization rates of neonatal gut microbiota were associated with the mode of delivery. Moreover, the colonization rates of *Bacteroides* and *Bifidobacterium* in the VD group were higher than those in the CS group. Also, the colonization rates of *Clostridium*, *Lactobacillus*, *Enterobacter*, *Streptococcus*, and *Enterococcus* in the CS group were higher than those in the VD group. The mode of delivery did not significantly affect the colonization rate of *Lactobacillus* during the 2nd week to the 1st month of life, the colonization rate of *Bifidobacterium* during the 4th to 6th month of life, the colonization rates of *Clostridium* and *Enterobacter* from the 7th month to the 1st year of life, and the colonization rates of *Clostridium*, *Bifidobacterium*, and *Enterococcus* after the 1st year of life. However, in the 1st week after delivery and the 2nd and 3rd months of life, the mode of delivery could affect the colonization rates of all the bacteria. In a systematic review, Rutayisire et al. found that during the first 3 months of life, the colonization rates of *Bifidobacterium* and *Bacteroides* were higher in the VD group, while the colonization rates of *Clostridium* and *Lactobacillus* were higher in the CS group [23], which were consistent with our results. In their study, during 6–12 months of life, the mode of delivery had less effect on the diversity and the colonization rates of *Bifidobacterium*, *Bacteroides*, *Clostridium*, and *Lactobacillus*; while in our study, during 6–12 months of life, the colonization rates of *Bifidobacterium* and *Bacteroides* in the VD group were greater than those in the CS group. This difference may be due to the experimental methods presented in the included studies. The findings of this study are consistent with the previous studies showing that the term CS-born infants lack the colonization of neonatal gut microbiota up to a year, with lower overall microbial diversity [6,38]. Another study demonstrated that a significant difference was observed between the CS and VD groups in terms of gut microbial colonization infants up to their 7th year of life [39]. Our study is in line with the study of Shao et al. (2019), suggesting that the mode of delivery can play an important factor in the diversity of neonatal gut microbiota, especially 4 days after birth. *Bifidobacterium*, *Bacteroides*, and *Parabacteroides* species in the samples from the VD group were more abundant than those in the CS group; while *Enterococcus*, *Staphylococcus epidermidis*, *Streptococcus*, *Klebsiella*, *Enterobacter cloacae*, and *Clostridium perfringens* species were observed in the premature babies delivered via CS in the hospital settings, and they also reported that other clinical factors such as prenatal antibiotic use, hospital stay, and breastfeeding had less effects [40]. In consistent with our results, the study conducted by Chee et al. in Singapore and Indonesia demonstrated that the colonization rate of *Lactobacillus* was higher in the CS group. Other studies have shown that the colonization rate of *Lactobacillus* in the VD group was significantly higher than that in the CS group [20,41]. In anal samples from the exposed infants and the VD-born infants, there is an early enrichment of *Lactobacillus* followed by a bloom of *Bacteroides* from week 2 that is not observed in infants not exposed to vaginal fluids [41]. In their study, Shao et al. demonstrated that no significant difference was observed between the two groups with respect to the colonization rate of *Lactobacillus* [40]. These differences may be attributed to techniques used for the analysis of the gut microbiota. Studies have also shown that demographic factors, including breastfeeding, age to stop breastfeeding, and antibiotic use, can affect the infant’s gut microbiota, but the mode of delivery would have the greatest effect on it [38]. Studies have shown that the incidence of diarrhea is inversely related to *Lactobacillus* and *Bifidobacterium* levels in children less than 5 years of age. Due to their possible beneficial effects on human health, *Lactobacillus* and *Bifidobacterium* as probiotic bacteria have also been used to prevent or reduce the risk of infant gastroenteritis [42,43]. An increase in the level of *Bifidobacterium* appears to play an important role in the development and maturation of the immune system, and increased levels of *Clostridium difficile* known as a nosocomial infection can cause gastroenteritis in infants [23]. Reduced levels of *Bifidobacterium* and increased *Clostridium* in CS-born infants may be due to antibiotic use [44]. Women undergoing CSs receive antibiotics before, during, and after delivery, especially during CS complications such as uterine rupture [45], bladder injury [46], etc., which may affect the gut microbiota diversity. Studies have shown that postnatal antibiotic use is associated with increased levels of *Clostridium* and decreased levels of *Bifidobacterium* and *Bacteroides*, and the use of antibiotics as a potential factor can affect the composition of gut microbiota [38,47]. Also, the reduced levels of bactericides were observed in infants born by VD or CS whose mother used antibiotic prophylaxis during delivery [6,40,44]. The lack of exposure to vaginal microbiota may be another possible reason for increased levels of Firmicutes species (*Lactobacillus*, *Staphylococcus*, *Streptococcus*, *Enterococcus*, and *Clostridium*) and reduced levels of *Bacteroides* among CS-born infants [23]. Previous studies have suggested that the *Bacteroides* may be transmitted from mother to child during birth [48,49]. Increased levels of *Clostridium* in CS-born infants may be attributed to nosocomial infections [45]. Breast milk contains the beneficial gut bacteria similar to probiotics, which can stimulate the growth of *Bifidobacterium* and *Lactobacillus* [51,52]. Infants born by CS are deprived of breast milk in the early stages, and there may be a reason for the decrease in these species in CS-born babies. Studies have demonstrated that formula feeding is associated with increased levels of *Clostridium difficile* and decreased *Bifidobacterium* [53,54]. A Danish cohort study showed that significant changes in the gut microbiota occurred, particularly from age 9 to 18 months, when cessation of breastfeeding and introduction of a complementary feeding induces replacement of a microbiota [55]. Although there are two theories about infant gut colonization: “sterile gut before birth” and “in uterus colonization hypothesis,” but the influence of the maternal microbiota on the organization of microbial population in uterus is yet to be determined [2]. Studies have shown that mother-to-baby transmission of bacteria occurs before birth and continues after birth [21,56]. During the third trimester of pregnancy, with the development of the nervous system, the fetus swallows a large amount of amniotic fluid, which causes the uterine microbiota to enter the baby’s digestive tract [57]. Recent studies have also shown that there are common bacteria between amniotic fluid and the meconium [58]. During the 1st week of life, the term infant gut is colonized by the Actinobacterial family (*Bifidobacterium*, *Propionibacterium*, *Corynebacterium*, and *Streptomyces*), Proteobacteria (*Ruminococcus*, *Enterobacter*, *Escherichia coli*, *Klebsiella*, and *Acinetobacter*), and Firmicutes (*Lactobacillus*, *Staphylococcus*, *Streptococcus*, *Enterococcus*, and *Clostridium*) [59,60]. Colonization can be altered by factors such as gestational age, delivery mode (VD or CS), formula feeding (breastfeeding or formula feeding), hygiene, and use of antibiotics. During the first 3 years of life, the environment and feeding have important roles in achieving an adult gut microbiome that affects the development of the immune and nervous systems. The human gut microbiota reaches the characteristics of an adult microbiota between the ages of 2 and 5 years [21]. In this study, all infants were born at term while studies have shown that the duration of pregnancy can also affect neonatal gut microbiota diversity. In preterm infants, there was a decrease in gut microbiota diversity and an increase in colonization rate of pathogenic organisms [61,62]. Compared to the term infants, premature infants have increases in anaerobes (such as *Enterococcus*, *Enterobacter*, *Lactobacillus*, and *Staphylococcus*) as well as decreases in the anaerobes (such as *Bifidobacterium*, *Bacteroides*, and *Atopobium*) [63,64]. In their study, Gregory et al. found that preterm infants born by VD had a higher rate of *Bacteroides* than CS-born infants, which is consistent with our results, indicating that the mode of delivery can affect both term and preterm infants.

Although there are higher complications with advanced maternal age [65], it has been shown that variables of maternal prenatal factors including geographic location, gestational hypertensive status, and maternal age did not affect the diversity of gut microbial taxa composition [66].

The mode of delivery affects the colonization of the neonatal gut microbiota. The colonization rates of *Bacteroides* and *Bifidobacterium* were lower in CS-born infants. Also, the colonization rates of *Clostridium*, *Lactobacillus*, *Enterobacter*, *Streptococcus*, *Enterococcus*, and *Staphylococcus* were higher in CS-born infants. The mode of delivery did not significantly affect the colonization rate of *Lactobacillus* during the 2nd week to the 1st month of life; the colonization rate of *Bifidobacterium* during the 4th to 6th month of life; the colonization rates of *Clostridium* and *Enterobacter* from the 7th month to the 1st year of life; and the colonization rates of *Clostridium*, *Bifidobacterium*, and *Enterococcus* after the 1st year of life.

The neonatal gut microbiota colonization seems to be important for health and development because the developing infant gut microbiome can influence metabolism, immune system function, and brain development [2]. The gut microbiota has three essential roles, namely, protective, metabolic, and trophic. Protective role includes prevention of the proliferation of pathogenic organisms; and the metabolic role includes the digestion and metabolism of milk and food in infants, the breakdown of toxins and drugs, vitamin synthesis, and ion absorption. Trophic role includes the growth and differentiation of the epithelial cells of the intestinal lumen, and the homeostatic maintenance of the immune system includes tolerance to food antigens [67,68]. The neonatal immune system will rapidly mature due to the influence of microbiota, diet, exposure to new microbes, and other environmental exposures [56,69].

The main message of this study for practitioners is that CS can cause many problems for infants and babies; moreover, it can change the pattern of infant’s gut microbiota, and hence, VD is the best method of delivery and we must avoid unnecessary and without medical indication CS. However, in the 1st week after delivery and the 2nd and 3rd months of life, the mode of delivery could affect the colonization rates of all the bacteria. Given that the mode of delivery affects the colonization of infant’s gut microbiota types, and the colonization of each type of microbiota has an effect on the baby’s growth, development, and health; therefore, it is recommended to conduct further studies in order to investigate the effect of the colonization type on delivery mode and on baby’s growth and development.

Some limitations of this study are as follows: failure to explore the microbiota types in most studies, a small number of studies that examined neonatal gut microbiota, and a small number of studies that examined the neonatal gut microbiota during 6 months of life, not mentioning the number of bacteria found in the infants gut and not distinguishing the type of bacteria found in the infant’s gut in terms of the mode of delivery and a small sample size.


**Ethics of approval:** This study has a code of ethics number IR.ABZUMS.REC.1399.168 from Alborz University of Medical Sciences.
